# Impact of a Systems-Based Intervention to Overcome Barriers to Ambulation and Improve Unit Culture

**DOI:** 10.1093/geroni/igaf122.3361

**Published:** 2025-12-31

**Authors:** David Tucker, Barbara King, Linsey Steege

**Affiliations:** University of Wisconsin–Madison, Madison, Wisconsin, United States; University of Wisconsin–Madison, Madison, Wisconsin, United States; University of Wisconsin–Madison School of Nursing, Madison, Wisconsin, United States

## Abstract

Low mobility of older adults during hospitalization results in devastating losses in functional ability by discharge. Nursing staff are identified as the primary healthcare provider responsible for maintaining patient functional status. However, nurses often lack knowledge and self-efficacy for assessing patient mobility and may not see patient ambulation as their role. Mobilizing Older adults Via a systems-based INtervention (MOVIN) is a multicomponent intervention designed to target system barriers and change in nurse behavior and ambulation culture on inpatient units. This presentation will report changes in nurse perceptions of barriers to ambulation and changes in unit ambulation culture pre/post MOVIN implementation. A cluster randomized controlled trial design was used to measure changes in nurse behavior and unit ambulation culture. Nursing staff (Registered Nurse (RN) and Certified Nursing Assistants (CNAs) from 2 hospitals and 4 inpatient adult medical units completed Barriers to Patient Ambulation and Ambulation Culture surveys. General Linear Modeling and propensity weights to adjust for covariant differences between pre and post were used as a function to determine group differences (pre/post) by units. Nursing staff completed 194 Pre and 169 Post surveys. RNs had a significant reduction in Barriers to Ambulation (knowledge, attitudes, behaviors) post-intervention. Across nursing staff, positive perceptions of Ambulation Culture increased post-intervention. Implementation of MOVIN was effective in reducing barriers to nurse ambulation and improving ambulation culture on hospital units. Addressing these barriers is critical to achieving sustained changes in nurse-initiated patient ambulation and improving functional outcomes for hospitalized older adults.

